# Interpretable machine learning for depression symptom classification in NHANES: Performance in a curated high-confidence corpus and the full survey population

**DOI:** 10.1016/j.ibneur.2026.08.009

**Published:** 2026-08-18

**Authors:** Omid Salimi, Zahra Ghahramani, Mahsa Taghavi Zenouz, Marziyeh Heidari, Alireza Amini, Fatemeh Nouroozi, Mobina Fathi, Arian Tavasol, Mohammad Reza Bateni

**Affiliations:** a Department of Medicine, Islamic Azad University, Najafabad, Iran; b Student research committee, Islamic Azad University, Najafabad, Iran; c Pharm.D. Student, Faculty of Pharmacy, Ardabil University of Medical Sciences, Ardabil, Iran; d School of Medicine, Tabriz university of Medical Sciences, Tabriz, Iran; e Faculty of Medicine, Hormozgan, University of Medical Sciences, Bandar Abbas, Iran; f Emergency Department of Sayad Shirazi Hospital, Golestan University of Medical Sciences, Gorgan, Iran; g Shahid Beheshti University of Medical Sciences, Tehran, Iran; h School of Medicine, Shahid Beheshti University of Medical Sciences, Tehran, Iran; i Computer Science Department, University of Tehran, Tehran, Iran

**Keywords:** Depression, Machine Learning, PHQ-9, NHANES, LightGBM, Random Forest, Mental Health, Predictive Modeling

## Abstract

**Background:**

Depressive disorders are among the most common psychiatric conditions worldwide and frequently remain undetected or misinterpreted. Scalable computational tools may help characterize depressive-symptom patterns in large health datasets, but their clinical use requires realistic validation and calibration.

**Objective:**

We evaluated machine-learning models for classifying PHQ-9 depressive-symptom status in NHANES and compared performance in a curated high-confidence corpus and the full analytic population.

**Methods:**

This cross-sectional analysis included 37,959 NHANES participants with valid PHQ-9 data and prespecified demographic, sleep, and dietary predictors. The primary outcome was mild-or-greater depressive symptoms, defined as PHQ-9 ≥ 5. A balanced high-confidence corpus was constructed using clear PHQ-9 rules, teacher-model confidence ranking, Isolation Forest filtering, and class-specific resampling. A LightGBM classifier was compared with logistic regression, random forest, gradient boosting, support vector machine, XGBoost, k-nearest neighbors, and Gaussian naïve Bayes. Performance, calibration, temporal validation, sensitivity analyses, and SHAP-based interpretability were assessed.

**Results:**

The curated corpus contained 5000 balanced observations. LightGBM achieved near-perfect curated-holdout performance, but full-population performance was moderate: accuracy 0.678, F1-score 0.503, precision 0.407, recall 0.659, and ROC-AUC 0.726. Comparator models showed similar full-population discrimination. Removing sleep predictors reduced ROC-AUC to 0.611. Full-population calibration was poor, and self-reported trouble sleeping was the dominant contributor.

**Conclusion:**

Interpretable machine learning identified reproducible depressive-symptom patterns in NHANES, largely driven by sleep variables. However, modest precision and poor calibration preclude stand-alone clinical use without external validation and recalibration.

## Introduction

Depression is among the most common and disabling mental health conditions worldwide, with more than 280 million people estimated to be affected and has been identified as a leading cause of disability across all age groups (GBD 2019 Mental Disorders Collaborators, 2022, World Health Organization, 2023). Heterogeneity in depressive symptoms, illness course, and treatment response complicates diagnosis, prognosis, and treatment planning. These challenges mean that depression is often underdiagnosed or misclassified in routine care, despite the widespread use of brief screening tools. Depression also involves interacting neurochemical, inflammatory, oxidative-stress, neuroendocrine, and neuroplasticity pathways, reinforcing its biological heterogeneity (Yarahmadi et al., 2026).

The Patient Health Questionnaire−9 (PHQ−9) has become one of the most commonly used and validated screening instruments for major depressive disorder in primary care and mental health settings (Manea et al., 2012). This nine-item self-report instrument is based on the Diagnostic and Statistical Manual of Mental Disorders (DSM-IV) criteria and asks the patient to quantify the frequency with which they experience a range of depressive symptoms over the last two weeks, including anhedonia, depressed mood, sleep disruption, fatigue, appetite changes, feelings of worthlessness, concentration difficulties, psychomotor symptoms, and suicidal ideation (Ford et al., 2020). The PHQ−9 is widely used for depressive-symptom screening and severity assessment, but it does not replace a structured clinical diagnostic evaluation (Manea et al., 2012, Kroenke et al., 2001).

Nevertheless, conventional approaches to depression assessment and monitoring have important limitations. Cross-sectional questionnaires such as the PHQ−9 capture symptoms at single time points and may fail to reflect longitudinal change, treatment response, or the dynamic interaction among symptom domains (Chekroud et al., 2016, Hong et al., 2021). Fixed cut-off scores, while pragmatic, do not fully account for individual differences in symptom trajectories or the heterogeneity of depressive illness, which ranges from episodic remissions and relapses to chronic, persistent courses (Fried and Nesse, 2015, Fried et al., 2017, Shatte et al., 2019).

Machine-learning methods offer adaptive modeling approaches that can capture complex and nonlinear relationships in clinical and population-based data (Scodari et al., 2023, Phillips et al., 2025). Compared to traditional statistics, gradient boosting and other tree-based methods can handle mixed data types, model nonlinear associations, and provide interpretable feature-level outputs, making them useful for structured survey-based prediction (Scodari et al., 2023, Coley et al., 2021, Nickson et al., 2023).

Recent studies have explored the use of machine learning models to predict depression from PHQ−9 scores and/or electronic health records, with the goal of enhancing clinician identification of patients with depressive symptoms and/or related risk factors (Scodari et al., 2023, Phillips et al., 2025, Nickson et al., 2023, Kim and Lee, 2021). However, the majority of this research uses a large number of high-dimensional data features, complex model architectures, and/or relatively inaccessible to clinicians with respect to their understanding of the meaning behind the model's outputs.

Therefore, this study used NHANES data and a small set of routinely available demographic, dietary, and sleep-related predictors to evaluate interpretable machine-learning models for PHQ−9 depressive-symptom classification. We specifically compared performance in a deliberately curated high-confidence corpus and in the full NHANES analytic population, assessed multiple comparator algorithms, examined calibration and threshold behavior, and evaluated robustness through sleep-variable exclusion, PHQ−9 ≥ 10 sensitivity analysis, alternative corpus-construction strategies, temporal holdout validation, and subgroup analyses.

## Materials and methods

### Data source and study population

We conducted a cross-sectional machine-learning analysis using data from the U.S. National Health and Nutrition Examination Survey (NHANES). NHANES is a large, publicly available health survey that combines questionnaire, examination, and laboratory data from the noninstitutionalized U.S. population (Curtin et al., 2013, Johnson et al., 2014). Participants were eligible if they had a valid Patient Health Questionnaire−9 (PHQ−9) total score and available data structure for the prespecified demographic, sleep, and dietary predictors. The dataset included NHANES survey cycles from 2005 to 2006 through the 2017-March 2020 pre-pandemic public release. The final analytic sample included 37,959 participants. Because the aim was predictive model development and internal/full-population performance assessment rather than population prevalence estimation, analyses were performed on the analytic dataset without applying NHANES survey weights. Baseline characteristics were summarized overall and stratified by PHQ−9 depressive-symptom status.

### Outcome definition

The PHQ−9 total score was used as the reference depressive-symptom measure. Scores range from 0 to 27, with higher values indicating greater depressive-symptom burden (Kroenke et al., 2001). The primary outcome was mild-or-greater depressive symptoms, defined as PHQ−9 total score ≥ 5. This threshold was selected to capture broad depressive-symptom positivity rather than probable major depressive disorder. A secondary sensitivity outcome was moderate-or-greater depressive symptoms, defined as PHQ−9 total score ≥ 10 (McIntyre et al., 2021). For high-confidence corpus construction, clear positive cases were defined as PHQ−9 total score > 5 and clear controls as PHQ−9 total score < 3. This design intentionally emphasized separable symptom groups for model development. The full NHANES analytic population, including participants outside these clear-case ranges, was then used to evaluate model performance under a more realistic distribution. Individual PHQ−9 items were not modeled separately; the PHQ−9 total score was used only to define depressive-symptom thresholds.

### Predictor variables

Predictors were selected to represent a small set of routinely available demographic, sleep, and dietary variables. Continuous predictors were age, usual sleep duration, total daily energy intake, and total daily fat intake. Categorical predictors were sex, educational attainment, self-reported trouble sleeping, and daytime sleepiness frequency. All raw variable names were mapped to standardized labels before analysis and reporting. Category codes were also translated into their true response labels; for example, sex was reported as male or female, self-reported trouble sleeping as yes or no, and daytime sleepiness frequency as never, rarely, sometimes, often, or almost always.

Variable-specific cleaning was applied before modeling. The PHQ−9 total score was restricted to the valid range of 0–27. Age and usual sleep duration were restricted to plausible values, and negative dietary intakes were treated as missing. Categorical refused and “don’t know” responses were set to missing where applicable. Missing categorical predictors were imputed using the most frequent category, and missing continuous predictors were imputed using the median. Categorical variables were one-hot encoded, and continuous variables were standardized. These transformations were implemented within scikit-learn pipelines to ensure that preprocessing was fitted only within the relevant training partitions.

### Descriptive analyses

Baseline characteristics were presented by PHQ−9 group: overall, PHQ−9 < 5, and PHQ−9 ≥ 5. Continuous variables were summarized as mean ± standard deviation. Categorical variables were summarized as n (%), with percentages calculated using nonmissing observations within each PHQ−9 group as the denominator. Between-group comparisons were descriptive. Welch *t*-tests were used for continuous variables, and chi-square tests or Fisher exact tests were used for categorical variables where appropriate. P values were not adjusted for multiple comparisons.

### High-confidence corpus construction

A balanced high-confidence corpus was created for model development. First, participants meeting the clear positive or clear control PHQ−9 criteria were identified. A preliminary teacher model was then used to rank records by classification confidence. The highest-confidence records were retained up to 2500 participants per class. Isolation Forest filtering was applied to reduce multivariate outliers. After filtering, class-specific resampling restored the development corpus to 5000 records, with 2500 positive and 2500 negative observations. This design was intended to create a separable development corpus for internal model training, not to estimate real-world screening performance. For this reason, the final model was also evaluated in the full NHANES analytic population, and sensitivity analyses tested alternative teacher and no-teacher sampling strategies.

### Model development and comparator algorithms

The primary classifier was a LightGBM gradient-boosted decision-tree model. The final model used a fixed reproducible configuration with 120 estimators, maximum depth of 8, 45 leaves, learning rate of 0.089, subsampling of 0.78, column subsampling of 0.92, L1 regularization of 0.045, L2 regularization of 0.087, and random seed 42. To evaluate whether performance was specific to LightGBM, comparator models were trained using the same predictor set and preprocessing framework. These included logistic regression, random forest, gradient boosting, k-nearest neighbors, Gaussian naïve Bayes, support vector machine, and XGBoost when available.

The curated corpus was split into training and holdout partitions. The training partition was further divided into inner training and validation subsets for threshold selection. The fitted model and selected operating threshold were then applied to both the curated holdout set and the full NHANES analytic population.

### Threshold selection and validation

Candidate probability thresholds from 0.05 to 0.95 were evaluated in increments of 0.05. The threshold maximizing F1-score in the validation split was selected for classification. To assess threshold stability, five-fold cross-validation was performed within the curated corpus, with inner validation-based threshold selection repeated in each fold. These thresholds were treated as exploratory operating points and not as clinical diagnostic cut-points. No operating threshold was selected on the final curated holdout set or during full NHANES analytic-population application.

### Model evaluation

Model performance was assessed using accuracy, F1-score, precision, recall, Matthews correlation coefficient, receiver-operating-characteristic area under the curve (ROC-AUC), and average precision. Bootstrap 95% confidence intervals were estimated using 1000 resamples. Confusion matrices were generated for the curated holdout set and the full NHANES population. Calibration was assessed using Brier score, expected calibration error, Hosmer-Lemeshow statistics, and calibration plots comparing predicted probabilities with observed event rates across risk deciles. These analyses were performed to distinguish discrimination from probability calibration.

### Sensitivity, robustness, and subgroup analyses

Several analyses were prespecified to evaluate robustness. First, all sleep-related predictors were removed to assess dependence on usual sleep duration, self-reported trouble sleeping, and daytime sleepiness frequency. Second, the outcome was redefined as PHQ−9 ≥ 10 to evaluate moderate-or-greater depressive symptoms. Third, alternative corpus-construction strategies were tested, including logistic-regression teacher selection and PHQ-rule sampling without teacher-model confidence ranking. Fourth, temporal holdout validation was performed by training on earlier NHANES survey cycles and evaluating on later cycles. Subgroup performance was examined across sex, age group, and educational attainment. Additionally, to evaluate whether teacher-model selection introduced algorithm-specific circularity, we repeated corpus construction using logistic-regression teacher selection and PHQ-rule sampling without teacher-model confidence ranking.

### Software and reproducibility

Model interpretation used native LightGBM feature importance, permutation importance based on ROC-AUC decrease, and SHAP analyses. Global SHAP values were computed for the primary LightGBM model using standardized feature labels. Calibration, threshold, subgroup, and interpretability outputs were exported as manuscript-ready Word tables and JPG figures. All analyses were conducted in Python version 3.13.7 with random seed 42 using pandas, NumPy, scikit-learn, LightGBM, XGBoost, SHAP, SciPy, matplotlib, and python-docx.

### Ethical considerations

NHANES protocols are reviewed and approved by the National Center for Health Statistics (NCHS) Research Ethics Review Board, and all participants provide written informed consent; for those under 18 years, parental or guardian consent is obtained. The present study was a secondary analysis of de‑identified, publicly available NHANES data and therefore did not require additional institutional review board approval or individual consent at the authors’ institutions. Reporting followed TRIPOD recommendations for prediction models and the STROBE guidelines for cross‑sectional studies.

## Results

### Analytic sample and depressive-symptom profile

**Fig. 1:**
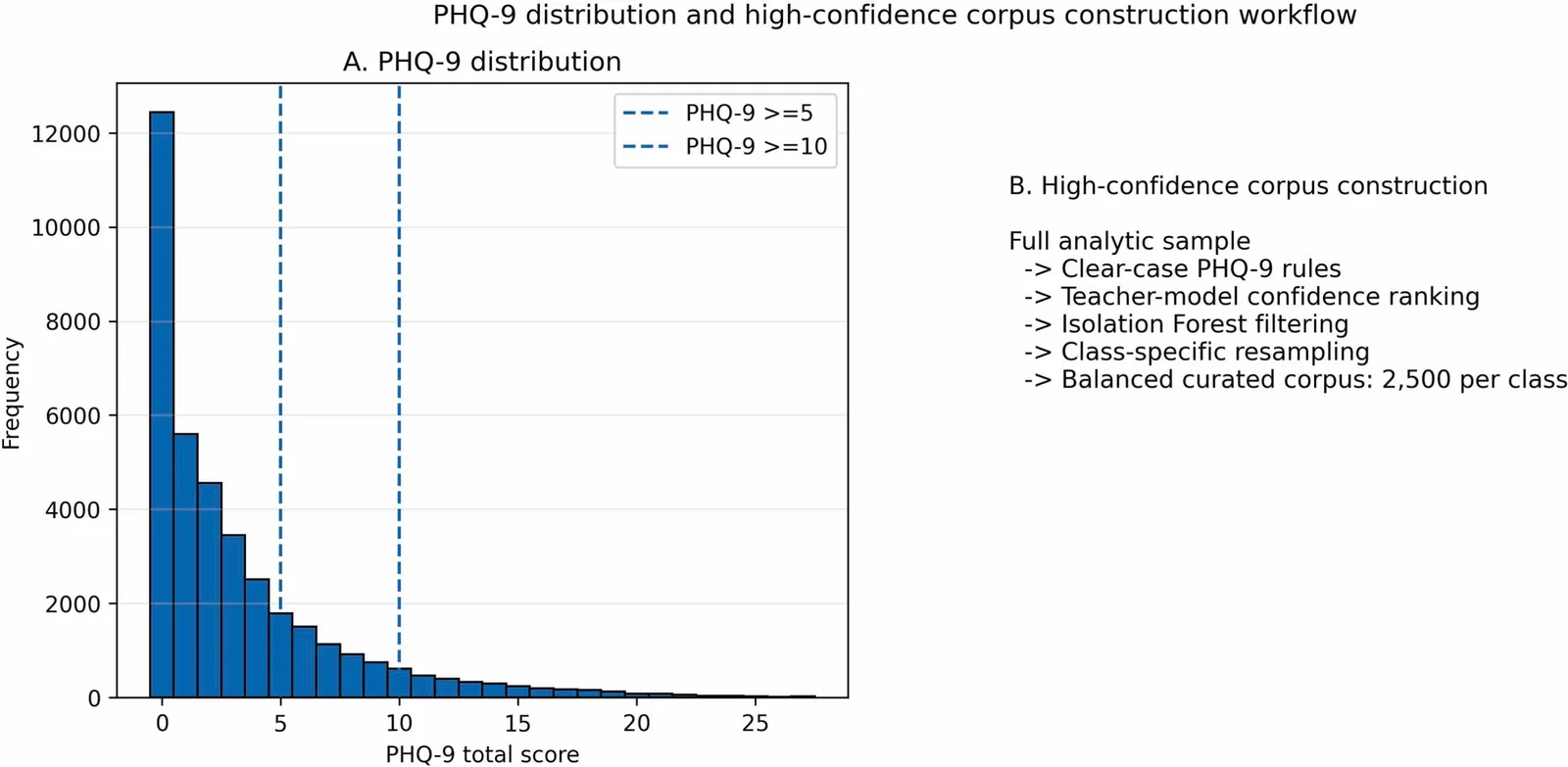
PHQ−9 distribution and high-confidence corpus construction workflow. Panel A shows the distribution of PHQ−9 total scores and indicates the PHQ−9 > =5 and PHQ−9 > =10 thresholds. Panel B summarizes construction of the balanced high-confidence corpus, including clear-case selection, teacher-model confidence ranking, Isolation Forest filtering, and class-specific resampling.

The final analytic sample included 37,959 NHANES participants, of whom 28,557 had PHQ−9 scores < 5 and 9402 met the primary threshold for mild-or-greater depressive symptoms (PHQ−9 ≥5). The PHQ−9 distribution was strongly right-skewed, with most participants reporting low symptom burden. Overall, the mean PHQ−9 score was 3.21 ± 4.25; it was 1.23 ± 1.35 among participants with PHQ−9 < 5 and 9.22 ± 4.40 among those with PHQ−9 ≥ 5 (Table 1; Fig. 1). Moderate-or-greater depressive symptoms, defined as PHQ−9 ≥ 10, were present in 3325 participants, representing 8.8% of the full analytic sample and 35.4% of the PHQ−9 ≥ 5 group.

**Table 1:** Baseline characteristics of the analytic sample stratified by PHQ−9 depressive-symptom status.

Characteristic	Overall	PHQ−9 < 5	PHQ−9 ≥ 5	P value
Participants, n	37959	28557	9402	
PHQ−9 total score, mean ± SD	3.21 ± 4.25	1.23 ± 1.35	9.22 ± 4.40	< 0.001
Age, years, mean ± SD	47.74 ± 18.70	47.91 ± 18.85	47.24 ± 18.23	0.002
Usual sleep duration, hours, mean ± SD	7.12 ± 1.54	7.19 ± 1.42	6.91 ± 1.85	< 0.001
Total daily energy intake, kcal, mean ± SD	2122.53 ± 1012.27	2130.49 ± 995.17	2098.35 ± 1062.23	0.010
Total daily fat intake, mean ± SD	81.58 ± 47.58	81.95 ± 47.22	80.44 ± 48.64	0.008
Moderate-or-greater depressive symptoms, PHQ−9 > =10, n (%)	3325 (8.8)	0 (0.0)	3325 (35.4)	< 0.001
Self-reported trouble sleeping: Yes, n (%)	9600 (25.3)	5219 (18.3)	4381 (46.6)	< 0.001
Self-reported trouble sleeping: No, n (%)	28349 (74.7)	23331 (81.7)	5018 (53.4)	
Daytime sleepiness frequency: Never, n (%)	5662 (25.0)	4953 (29.1)	709 (12.7)	< 0.001
Daytime sleepiness frequency: Rarely: 1 time per month, n (%)	5098 (22.5)	4319 (25.3)	779 (13.9)	
Daytime sleepiness frequency: Sometimes: 2–4 times per month, n (%)	6821 (30.1)	5162 (30.3)	1659 (29.7)	
Daytime sleepiness frequency: Often: 5–15 times per month, n (%)	3407 (15.1)	1940 (11.4)	1467 (26.3)	
Daytime sleepiness frequency: Almost always: 16–30 times per month, n (%)	1643 (7.3)	672 (3.9)	971 (17.4)	
Sex: Male, n (%)	18697 (49.3)	14933 (52.3)	3764 (40.0)	< 0.001
Sex: Female, n (%)	19262 (50.7)	13624 (47.7)	5638 (60.0)	
Educational attainment: Less than 9th grade, n (%)	3403 (9.0)	2394 (8.4)	1009 (10.7)	< 0.001
Educational attainment: 9th−11th grade, including 12th grade without diploma, n (%)	4936 (13.0)	3359 (11.8)	1577 (16.8)	
Educational attainment: High school graduate/GED or equivalent, n (%)	8305 (21.9)	6097 (21.4)	2208 (23.5)	
Educational attainment: Some college or associate degree, n (%)	10791 (28.4)	8041 (28.2)	2750 (29.2)	
Educational attainment: College graduate or above, n (%)	8298 (21.9)	6997 (24.5)	1301 (13.8)	
Educational attainment: Missing or unavailable, n (%)	2226 (5.9)	1669 (5.8)	557 (5.9)	

Values are presented as mean ± standard deviation for continuous variables and n (%) for categorical variables. Percentages for categorical variables are calculated using non-missing observations within each PHQ−9 group as the denominator. P values compare PHQ−9 < 5 versus PHQ−9 ≥ 5 groups using Welch *t*-tests for continuous variables and chi-square tests or Fisher exact tests for categorical variables. P values are descriptive and were not adjusted for multiple comparisons. The primary outcome was mild-or-greater depressive symptoms, defined as PHQ−9 total score ≥ 5.

Participants with PHQ−9 ≥ 5 differed from those with lower symptom scores across several clinically relevant characteristics. Usual sleep duration was shorter in the PHQ−9 ≥ 5 group than in the PHQ−9 < 5 group (6.91 ± 1.85 versus 7.19 ± 1.42 h; p < 0.001). Self-reported trouble sleeping was also more than twice as common in the PHQ−9 ≥ 5 group (46.6% versus 18.3%; p < 0.001). Daytime sleepiness showed a graded pattern: participants with PHQ−9 ≥ 5 were less likely to report never feeling overly sleepy during the day and more likely to report often or almost always experiencing daytime sleepiness. Female participants were overrepresented in the PHQ−9 ≥ 5 group, and educational attainment differed significantly by PHQ−9 status (Table 1). Standardized variable definitions and observed category frequencies are provided in Tables S1 and S6, while PHQ−9 distribution, outcome prevalence, and missingness after variable-specific cleaning are summarized in Table S7.

### Primary model development and full-population performance

The high-confidence corpus was built from 30,215 clear-case candidate records, including 7617 candidate positives and 22,598 candidate controls. After teacher-model confidence ranking, Isolation Forest filtering, and class-specific resampling, the final curated corpus contained 5000 observations, balanced as 2500 positives and 2500 negatives (Table 2; Fig. 1).

**Table 2:** Primary model performance in the curated holdout set and full NHANES population (validation-selected threshold).

Analysis	Accuracy	F1-score	Precision	Recall	MCC	ROC-AUC	Average precision
Curated holdout set	0.9990	0.9990	0.9980	1.0000	0.9980	1.0000	1.0000
Full NHANES population	0.6776	0.5031	0.4069	0.6589	0.3018	0.7260	0.4745

**Fig. 2:**
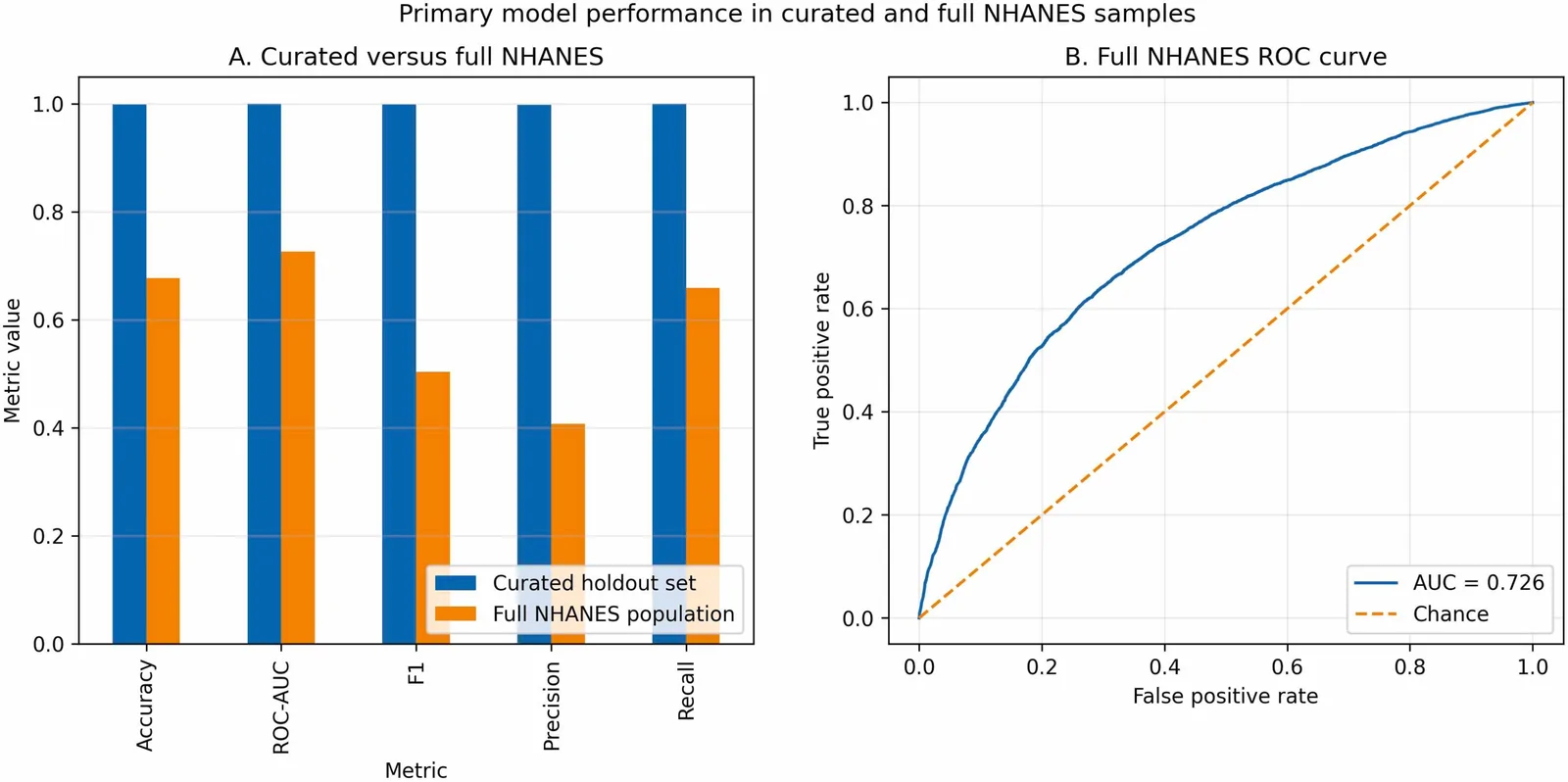
Primary model performance in the curated holdout set and full NHANES population. Panel A compares classification metrics between the high-confidence curated holdout set and the full NHANES analytic population. Panel B shows the receiver-operating-characteristic curve for the full NHANES population.

In the curated holdout set, the primary LightGBM model achieved near-perfect internal performance, with accuracy 0.999, F1-score 0.999, precision 0.998, recall 1.000, Matthews correlation coefficient 0.998, ROC-AUC 1.000, and average precision 1.000 (Table 2; Fig. 2). When applied to the full NHANES analytic population, performance attenuated substantially. Full-population accuracy was 0.678, F1-score 0.503, precision 0.407, recall 0.659, Matthews correlation coefficient 0.302, ROC-AUC 0.726, and average precision 0.475. Bootstrap intervals were narrow, with full-population ROC-AUC 0.720–0.732, F1-score 0.496–0.511, precision 0.400–0.415, and recall 0.650–0.669 (Table 2). This contrast indicates that the curated corpus was highly separable, whereas full-population classification was more modest. Unlike the curated corpus, the full NHANES evaluation retained the complete PHQ−9 distribution among eligible participants, including borderline scores.

### Comparator models and threshold behavior

Full-population discrimination was broadly similar across several model classes (Table 3). Random forest and gradient boosting produced the highest ROC-AUC values, both approximately 0.729, followed closely by support vector machine (0.728), the primary gradient-boosting model (0.726), logistic regression (0.726), and XGBoost (0.725). K-nearest neighbors and Gaussian naïve Bayes had lower ROC-AUC values of 0.707 and 0.689, respectively. The complete curated-holdout and full-population model comparison is shown in Table S2, where most models displayed near-perfect curated-holdout performance but converged to moderate discrimination in the full NHANES population. Cross-validated threshold analyses showed stable internal threshold behavior in the curated corpus, with fold-level performance remaining near ceiling; these thresholds were treated as internal operating points rather than clinical cut-points (Figure S5).

**Table 3:** Full-population performance of machine-learning and statistical comparator models.

Model	Accuracy	F1-score	Precision	Recall	MCC	ROC-AUC	Average precision
Primary gradient boosting model	0.6968	0.5030	0.4234	0.6195	0.3069	0.7259	0.4735
Logistic regression	0.6922	0.5014	0.4187	0.6247	0.3033	0.7257	0.4814
Random forest	0.6987	0.5050	0.4258	0.6204	0.3100	0.7290	0.4641
Gradient boosting	0.7047	0.5050	0.4318	0.6082	0.3122	0.7289	0.4845
Support vector machine	0.6703	0.5019	0.4010	0.6706	0.2987	0.7284	0.4819
XGBoost	0.7019	0.5036	0.4286	0.6103	0.3093	0.7251	0.4687
K-nearest neighbors	0.7202	0.4996	0.4485	0.5638	0.3128	0.7065	0.4186
Gaussian naive Bayes	0.6820	0.4879	0.4058	0.6115	0.2827	0.6885	0.3767

This table compares discrimination and classification performance across the primary gradient-boosting model and comparator algorithms in the full NHANES analytic population. MCC denotes Matthews correlation coefficient; ROC-AUC denotes area under the receiver-operating-characteristic curve.

Classification metrics in this table were calculated using a common 0.50 probability threshold for model comparison. The primary operating-point metrics in Table 2, Table 4 used the validation-selected threshold.

### Sensitivity and robustness analyses

**Fig. 3:**
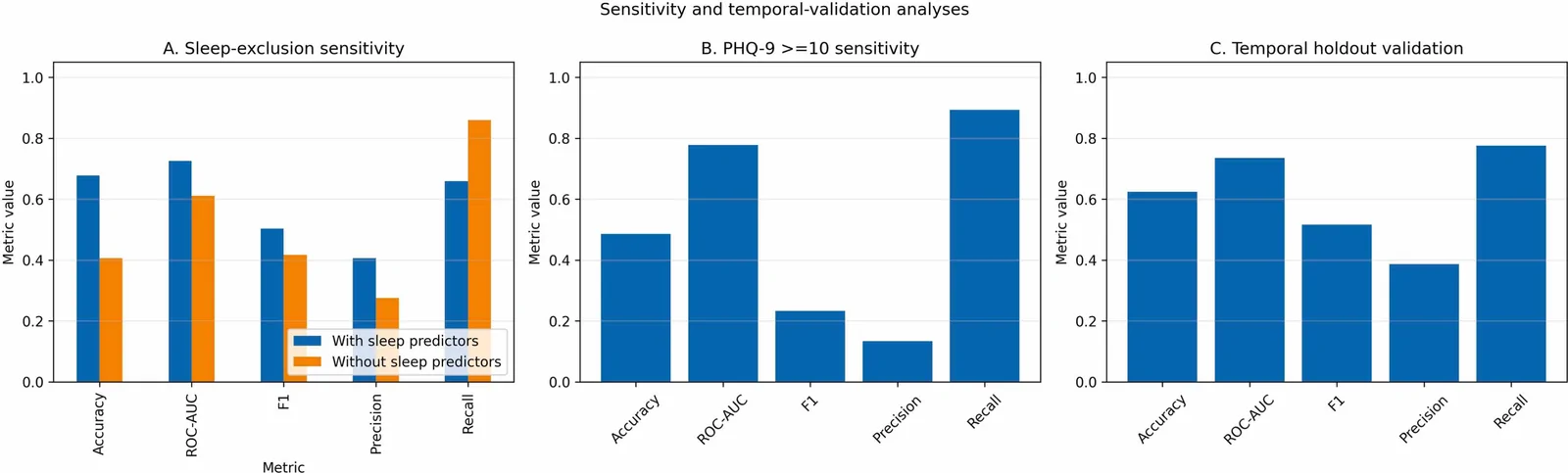
Sensitivity and temporal-validation analyses. Panel A compares full-population performance before and after exclusion of sleep-related predictors. Panel B shows performance when the outcome was redefined as PHQ−9 > =10. Panel C shows temporal holdout validation after training on earlier NHANES cycles and evaluating on later cycles.

Sensitivity analyses clarified the source and stability of the predictive signal (Table 4; Fig. 3). Sensitivity analysis was used to examine potential conceptual overlap between sleep predictors and PHQ−9 symptom content. Removing sleep-related predictors substantially reduced full-population discrimination: ROC-AUC decreased from 0.726 to 0.611, precision from 0.407 to 0.276, and average precision from 0.475 to 0.324, while recall increased to 0.860. This pattern indicates that sleep-related variables carried a large share of the model’s signal.

**Table 4:** Sensitivity, robustness, and temporal-validation analyses.

Analysis	Accuracy	F1-score	Precision	Recall	MCC	ROC-AUC	Average precision
Primary model, PHQ−9 > =5, full NHANES population	0.6776	0.5031	0.4069	0.6589	0.3018	0.7260	0.4745
Excluding sleep-related predictors, PHQ−9 > =5	0.4062	0.4176	0.2758	0.8596	0.1199	0.6112	0.3239
Sensitivity outcome, PHQ−9 > =10	0.4858	0.2333	0.1342	0.8932	0.1949	0.7785	0.2483
Logistic-regression teacher selection	0.7103	0.4965	0.4360	0.5767	0.3045	0.7138	0.4429
No teacher; PHQ-rule sampling only	0.6377	0.4970	0.3787	0.7226	0.2873	0.7259	0.4659
Temporal holdout validation	0.6246	0.5163	0.3868	0.7761	0.3048	0.7352	0.4783

This table summarizes model performance under alternative analytic conditions, including exclusion of sleep-related predictors, use of PHQ−9 > =10 as a more clinically specific outcome, alternative corpus-construction strategies, and temporal holdout validation. MCC denotes Matthews correlation coefficient; ROC-AUC denotes area under the receiver-operating-characteristic curve.

When the outcome was redefined as moderate-or-greater depressive symptoms using PHQ−9 ≥ 10, ROC-AUC increased to 0.779 and recall reached 0.893, but precision fell to 0.134 and F1-score to 0.233 (Table 4; Fig. 3). Thus, the model ranked participants with higher symptom burden reasonably well but generated many false-positive classifications under the stricter outcome definition. Alternative corpus-construction strategies yielded similar full-population discrimination. Logistic-regression teacher selection produced ROC-AUC 0.714, whereas PHQ-rule sampling without teacher-model confidence ranking produced ROC-AUC 0.726. Temporal holdout validation also supported reproducible moderate discrimination across survey cycles, with ROC-AUC 0.735, recall 0.776, precision 0.387, and average precision 0.478 (Table 4; Fig. 3).

### Interpretability, calibration, and subgroup performance

**Fig. 4:**
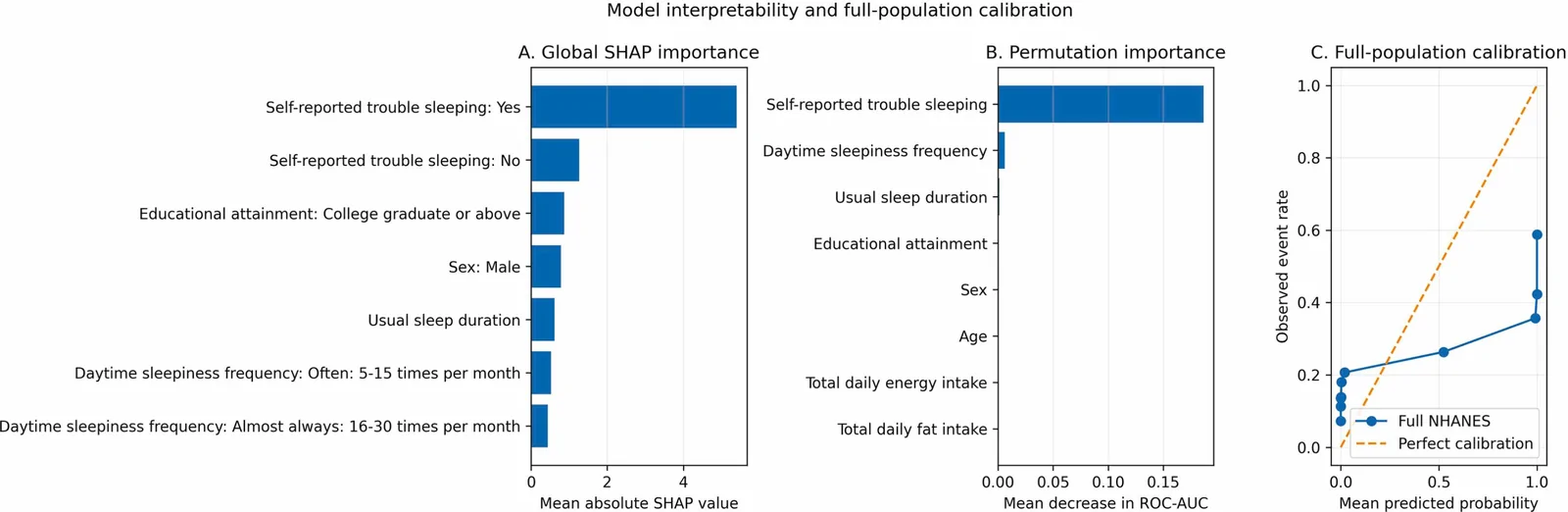
Model interpretability and full-population calibration. Panel A shows global SHAP feature contributions when available, otherwise native model feature importance. Panel B summarizes permutation importance for the original predictors. Panel C shows the full NHANES calibration plot comparing mean predicted probabilities with observed event rates across risk deciles.

Interpretability analyses consistently highlighted sleep-related predictors (Fig. 4). Global SHAP importance identified self-reported trouble sleeping, especially the “Yes” category, as the dominant feature. Permutation importance showed a similar pattern: permuting self-reported trouble sleeping produced the largest decrease in ROC-AUC, followed by daytime sleepiness frequency and usual sleep duration. Native LightGBM importance ranked usual sleep duration, total daily fat intake, age, daytime sleepiness, and self-reported trouble sleeping among the highest-ranking features (Table S3; Figure S1). The SHAP dependence plot further showed that the presence of self-reported trouble sleeping contributed strongly toward classification as PHQ−9 ≥ 5 (Figure S2). A local SHAP explanation for the highest predicted-risk false-positive case is provided in Figure S3. Sleep-related predictors, especially self-reported trouble sleeping and almost-always daytime sleepiness, strongly increased the model output despite PHQ−9 < 5, illustrating how the dominant sleep signal may contribute to false-positive classification.

Calibration analyses revealed a marked difference between curated and full-population settings. In the curated holdout set, calibration was nearly perfect, with Brier score 0.0000, expected calibration error 0.0003, and Hosmer-Lemeshow p = 1.000. In the full NHANES population, however, calibration was poor, with Brier score 0.2816, expected calibration error 0.2797, and Hosmer-Lemeshow p < 0.001 (Table 5; Fig. 4). Calibration deciles confirmed that observed event rates diverged substantially from predicted probabilities in the full population, particularly at high predicted-risk levels (Table S4). The full-population confusion matrix showed 6195 true positives, 19,527 true negatives, 9030 false positives, and 3207 false negatives (Table 5; Figure S4). Subgroup analyses showed broadly similar discrimination by sex and age, with ROC-AUC 0.718 in males, 0.719 in females, 0.708 among participants < 40 years, 0.746 among those aged 40–59 years, and 0.724 among those ≥ 60 years. Educational-attainment subgroup ROC-AUC values ranged from 0.662 to 0.723 (Table S5). Overall, the model showed reproducible moderate ranking discrimination in the full population, but limited precision and poor calibration argue against interpreting raw predicted probabilities as calibrated clinical risks.

**Table 5:** Calibration and classification error profile in the curated holdout set and full NHANES population.

Sample	Brier score	ECE	Hosmer-Lemeshow chi-square	df	p-value	TP	TN	FP	FN
Curated holdout set	0.0000	0.0003	0.3492	8	1.000	500	499	1	0
Full NHANES population	0.2816	0.2797	14493058.1946	8	< 0.001	6195	19527	9030	3207

This table reports calibration metrics and confusion-matrix counts. Calibration and error profiles are shown to contextualize model discrimination and avoid overinterpreting raw predicted probabilities as calibrated clinical risks. ECE denotes expected calibration error. TP: true positive; TN: true negative; FP: false positive; FN: false negative.

## Discussion

In this NHANES-based machine-learning analysis, we found that depression symptom classification was highly separable in a deliberately curated high-confidence corpus but substantially more challenging in the full survey population. The primary LightGBM model achieved near-perfect performance in the balanced curated holdout set; however, when applied to the full NHANES analytic sample, discrimination attenuated to a moderate level, with modest precision and poor probability calibration. This contrast is central to the interpretation of our findings. It indicates that high internal performance reflected, at least in part, the separability introduced by clear-case sampling rather than direct readiness for population-level clinical screening. Across comparator models, full-population performance was broadly similar, suggesting that the achievable signal was not unique to one algorithm. Sleep-related variables, particularly self-reported trouble sleeping, carried the dominant predictive information, while sensitivity analyses showed that removing sleep features markedly reduced model performance. Together, these findings support the value of interpretable machine-learning approaches for characterizing depressive-symptom patterns, but they also emphasize the need for cautious deployment, calibration, and external validation before clinical use. These findings are consistent with prior work using objective or digital behavioral measures, which has linked disrupted sleep and daily activity patterns with depressive-symptom burden (Albu et al., 2019, Maatoug et al., 2022).

These results show that interpretable machine learning can characterize depressive-symptom patterns in large survey data, but they do not establish a deployment-ready clinical screening tool. More broadly, prediction-modeling studies emphasize the importance of calibration assessment, conventional comparator analyses, and independent replication before translating flexible models into practice (Plessen et al., 2025, Baadkoube Hazaveh et al., 2026, Garriga et al., 2022). The similarity between LightGBM and comparator models suggests that the available predictors carried a reproducible but limited signal, rather than a uniquely algorithm-specific advantage.

Our findings should be interpreted within the broader literature on PHQ−9 measurement, depression heterogeneity, and the translational limits of predictive modeling. The PHQ−9 is a brief, validated measure of depressive-symptom severity, but its operating characteristics depend on the selected threshold and clinical context; prior validation and meta-analytic work support its usefulness while cautioning against rigid interpretation of any single cut-off (Manea et al., 2012, Kroenke et al., 2001). This issue is especially relevant for our study because the primary outcome was PHQ−9 ≥ 5, which captures mild-or-greater depressive symptoms rather than probable major depressive disorder. Depression itself is also phenotypically heterogeneous. Fried and Nesse showed extensive variation in symptom profiles among individuals meeting criteria for major depression, suggesting that total-score thresholds can obscure clinically meaningful differences in symptom composition (Fried and Nesse, 2015). This heterogeneity helps explain why classification was nearly perfect in our curated high-confidence corpus but only moderate when applied to the full NHANES distribution.

Our results also align with a growing but mixed machine-learning literature in depression. Chekroud et al. demonstrated that patient-reported clinical-trial data could predict antidepressant remission above chance, but performance declined when the model was externally tested (Chekroud et al., 2016). Similarly, a systematic review of EHR-based depression prediction found that reported AUC values were often promising, although regression methods could perform as well as more complex machine-learning models and concerns about generalizability and interpretability remained (Nickson et al., 2023). Nemesure et al. also reported only moderate performance for detecting depression from nonpsychiatric EHR-derived features, supporting the view that accessible structured predictors contain signal but not enough for definitive classification (Nemesure et al., 2021). Coley et al. provided an important cautionary counterpoint: even rich EHR data and advanced models poorly predicted psychotherapy outcomes and showed poor calibration (Coley et al., 2021). In this context, our full-population ROC-AUC of 0.726 and poor calibration should be viewed as realistic rather than disappointing.

Recent NHANES-based machine-learning studies further support the feasibility of explainable depression prediction. Vu et al. used multiple supervised models and SHAP analysis to identify demographic, socioeconomic, and health-related predictors of PHQ−9 ≥ 10 depression in NHANES (Vu et al., 2025). Xie et al., focusing on adults with obesity, reported that an ensemble model achieved strong discrimination and identified sleep disturbance, poverty-income ratio, and sex as leading predictors (Xie et al., 2026). Our study extends this literature by explicitly contrasting curated-corpus performance with full-population performance, adding calibration assessment, sleep-exclusion sensitivity, and alternative corpus-construction analyses.

The dominance of sleep-related variables in our model is biologically and epidemiologically plausible. A prospective meta-analysis found that insomnia was associated with more than a twofold increased risk of subsequent depression (Li et al., 2016). Longitudinal evidence also indicates a bidirectional relation between sleep and internalizing symptoms, suggesting that sleep problems may be both a marker and a contributor to depressive symptom burden (Bacaro et al., 2024). Digital phenotyping work has similarly shown that wearable-derived sleep and movement features can moderately detect depression symptom variability over time (Price et al., 2023). In our analysis, self-reported trouble sleeping was the strongest predictor, and removing sleep variables reduced ROC-AUC from 0.726 to 0.611. Thus, the model captured a robust and well-established sleep-depression signal, but this signal should be interpreted as depressive-symptom characterization rather than independent diagnostic discovery.

Future work should extend these results in several directions: validating the model prospectively and in independent cohorts; examining longitudinal PHQ‑9 patterns and temporal dynamics of sleep and mood; integrating additional clinical, behavioral, or multimodal data sources; and embedding such models into real‑world care pathways or public health surveillance systems. These steps will be essential to more precisely quantify clinical and public health impact and to determine whether such models can improve symptom monitoring, referral prioritization, or population-level mental health surveillance.

## Limitations

This study has several limitations that should be considered when interpreting the findings. First, the cross‑sectional structure of NHANES precludes causal inference between predictors and depressive symptoms; associations should therefore be viewed as descriptive and predictive rather than etiologic. Second, although NHANES is designed as a population-based survey, the present modeling analysis was unweighted and should not be interpreted as producing nationally representative prevalence estimates. Third, we restricted our models to eight routinely available predictors, selected for completeness across NHANES cycles, which likely omits important psychological, clinical, or social determinants of depression. Fourth, although the PHQ‑9 cut‑off of ≥ 5 is widely used, it does not fully capture the heterogeneity of depressive presentations, and our high‑confidence sampling strategy (PHQ‑9 >5 vs <3) further departs from the true population distribution. This manual construction of a balanced, curated corpus improves internal performance but limits ecological validity, as reflected in the more modest metrics observed when the model was applied to the full NHANES distribution. Finally, although temporal holdout validation across NHANES cycles supported reproducible moderate discrimination, all analyses still used NHANES data; external validation in independent datasets and clinical settings remains necessary.

## Conclusion

In this NHANES-based machine-learning study, an interpretable LightGBM model showed near-perfect discrimination within a deliberately curated high-confidence corpus but only moderate discrimination when applied to the full survey population. This divergence underscores the importance of evaluating predictive models beyond artificially separable development samples. Sleep-related variables, particularly self-reported trouble sleeping, contributed strongly to classification, and sensitivity analyses confirmed that model performance was substantially attenuated after their removal. Although temporal validation and comparator-model analyses supported a reproducible predictive signal, full-population precision was limited and calibration was poor. Therefore, the model should not be interpreted as a deployment-ready diagnostic tool or as a source of calibrated individual risk estimates. Rather, these findings support the use of transparent machine-learning approaches to characterize depressive-symptom patterns in large health surveys and to generate hypotheses for future screening frameworks. External validation, recalibration, and prospective clinical evaluation are required before any clinical or public health implementation.

## Compliance with ethical standards

This study utilized publicly available, fully anonymized data from the National Health and Nutrition Examination Survey (NHANES). The NHANES survey protocol was approved by the National Center for Health Statistics Research Ethics Review Board. All adult participants provided written informed consent, and for participants under 18 years, consent was obtained from a parent or guardian. The study was conducted in accordance with the ethical standards of the relevant institutional and national research committee.

## RCT registration code

Not applicable.

## Authors contribution

All authors contributed significantly to this work and met ICMJE criteria of authorship. All authors read and approved the final manuscript.

## CRediT authorship contribution statement

**Arian Tavasol:** Writing – review & editing, Validation, Supervision, Methodology. **Mohammad Reza Bateni:** Writing – review & editing, Supervision. **Fatemeh Nouroozi:** Writing – review & editing, Formal analysis, Data curation. **Mobina Fathi:** Writing – review & editing, Validation, Supervision, Methodology. **Alireza Amini:** Writing – review & editing, Writing – original draft. **Mahsa Taghavi Zenouz:** Writing – review & editing, Writing – original draft. **Marziyeh Heidari:** Writing – review & editing, Writing – original draft. **Omid Salimi:** Writing – review & editing, Writing – original draft, Methodology. **Zahra Ghahramani:** Writing – review & editing, Writing – original draft.

## Consent to participate

Not applicable for this secondary analysis of de-identified public data; written informed consent was obtained by NHANES during the original survey.

## Declaration of Generative AI and AI-assisted technologies in the writing process

During the preparation of this manuscript DeepSeek-R1 was used to improve readability and language.

## Ethics and consent to publish declaration

NHANES protocols were approved by the NCHS Research Ethics Review Board, and participants provided written informed consent. This secondary analysis used de-identified public data and did not require additional local IRB approval.

## Funding

None.

## Declaration of Competing Interest

The authors declare that they have no financial or personal relationships that could inappropriately influence or bias the content of this work.

## Data Availability

NHANES data are publicly available through the National Center for Health Statistics. The analytic code and reproducibility materials are available from the corresponding author upon reasonable request.

## Footnotes

Appendix A Supplementary data associated with this article can be found in the online version at doi:10.1016/j.ibneur.2026.08.009.
