# Supplementary material for: Interpretable machine learning for depression symptom classification in NHANES: Performance in a curated high-confidence corpus and the full survey population
Source: IBRO Neurosci Rep. 2026 Aug 18;21:738–46. doi: 10.1016/j.ibneur.2026.08.009 (PMC13563247; doi:10.1016/j.ibneur.2026.08.009)
Supplement: Supplementary file 1 [file mmc1.docx]

**Table S1.** Standardized variable dictionary used for analysis and reporting.

| Raw variable | Standardized label | Definition used in analysis |
| --- | --- | --- |
| sum | PHQ-9 total score | Total depressive-symptom severity score ranging from 0 to 27. |
| binary_target | Mild-or-greater depressive symptoms | Primary binary outcome defined as PHQ-9 total score ≥5. |
| binary_target_phq10 | Moderate-or-greater depressive symptoms | Sensitivity outcome defined as PHQ-9 total score ≥10. |
| sleep_hours | Usual sleep duration | Self-reported usual sleep duration in hours. |
| slq050 | Self-reported trouble sleeping | Participant report of having told a doctor or health professional about trouble sleeping. |
| slq120 | Daytime sleepiness frequency | Frequency of feeling overly sleepy during the day in the past month. |
| riagendr | Sex | Participant sex as recorded in NHANES demographic data. |
| ridageyr | Age | Participant age in years. |
| dmdeduc2 | Educational attainment | Highest educational level reported by adult participants. |
| dr1ikcal | Total daily energy intake | Day-1 total kilocalorie intake from NHANES 24-hour dietary recall. |
| dr1itfat | Total daily fat intake | Day-1 total fat intake from NHANES 24-hour dietary recall. |
| sddsrvyr | NHANES survey cycle | Survey-cycle indicator used for temporal validation. |
| seqn | Participant identifier | NHANES respondent sequence number. |

**Table S2.** Complete performance comparison across all evaluated models.

| Model | Sample | Metric | Value |
| --- | --- | --- | --- |
| Primary gradient boosting model | Curated holdout set | Accuracy | 1.0000 |
| Primary gradient boosting model | Curated holdout set | F1-score | 1.0000 |
| Primary gradient boosting model | Curated holdout set | Precision | 1.0000 |
| Primary gradient boosting model | Curated holdout set | Recall | 1.0000 |
| Primary gradient boosting model | Curated holdout set | Matthews correlation coefficient | 1.0000 |
| Primary gradient boosting model | Curated holdout set | Area under the ROC curve | 1.0000 |
| Primary gradient boosting model | Curated holdout set | Average precision | 1.0000 |
| Primary gradient boosting model | Full NHANES population | Accuracy | 0.6968 |
| Primary gradient boosting model | Full NHANES population | F1-score | 0.5030 |
| Primary gradient boosting model | Full NHANES population | Precision | 0.4234 |
| Primary gradient boosting model | Full NHANES population | Recall | 0.6195 |
| Primary gradient boosting model | Full NHANES population | Matthews correlation coefficient | 0.3069 |
| Primary gradient boosting model | Full NHANES population | Area under the ROC curve | 0.7259 |
| Primary gradient boosting model | Full NHANES population | Average precision | 0.4735 |
| Logistic regression | Curated holdout set | Accuracy | 0.9990 |
| Logistic regression | Curated holdout set | F1-score | 0.9990 |
| Logistic regression | Curated holdout set | Precision | 1.0000 |
| Logistic regression | Curated holdout set | Recall | 0.9980 |
| Logistic regression | Curated holdout set | Matthews correlation coefficient | 0.9980 |
| Logistic regression | Curated holdout set | Area under the ROC curve | 1.0000 |
| Logistic regression | Curated holdout set | Average precision | 1.0000 |
| Logistic regression | Full NHANES population | Accuracy | 0.6922 |
| Logistic regression | Full NHANES population | F1-score | 0.5014 |
| Logistic regression | Full NHANES population | Precision | 0.4187 |
| Logistic regression | Full NHANES population | Recall | 0.6247 |
| Logistic regression | Full NHANES population | Matthews correlation coefficient | 0.3033 |
| Logistic regression | Full NHANES population | Area under the ROC curve | 0.7257 |
| Logistic regression | Full NHANES population | Average precision | 0.4814 |
| Random forest | Curated holdout set | Accuracy | 1.0000 |
| Random forest | Curated holdout set | F1-score | 1.0000 |
| Random forest | Curated holdout set | Precision | 1.0000 |
| Random forest | Curated holdout set | Recall | 1.0000 |
| Random forest | Curated holdout set | Matthews correlation coefficient | 1.0000 |
| Random forest | Curated holdout set | Area under the ROC curve | 1.0000 |
| Random forest | Curated holdout set | Average precision | 1.0000 |
| Random forest | Full NHANES population | Accuracy | 0.6987 |
| Random forest | Full NHANES population | F1-score | 0.5050 |
| Random forest | Full NHANES population | Precision | 0.4258 |
| Random forest | Full NHANES population | Recall | 0.6204 |
| Random forest | Full NHANES population | Matthews correlation coefficient | 0.3100 |
| Random forest | Full NHANES population | Area under the ROC curve | 0.7290 |
| Random forest | Full NHANES population | Average precision | 0.4641 |
| Gradient boosting | Curated holdout set | Accuracy | 1.0000 |
| Gradient boosting | Curated holdout set | F1-score | 1.0000 |
| Gradient boosting | Curated holdout set | Precision | 1.0000 |
| Gradient boosting | Curated holdout set | Recall | 1.0000 |
| Gradient boosting | Curated holdout set | Matthews correlation coefficient | 1.0000 |
| Gradient boosting | Curated holdout set | Area under the ROC curve | 1.0000 |
| Gradient boosting | Curated holdout set | Average precision | 1.0000 |
| Gradient boosting | Full NHANES population | Accuracy | 0.7047 |
| Gradient boosting | Full NHANES population | F1-score | 0.5050 |
| Gradient boosting | Full NHANES population | Precision | 0.4318 |
| Gradient boosting | Full NHANES population | Recall | 0.6082 |
| Gradient boosting | Full NHANES population | Matthews correlation coefficient | 0.3122 |
| Gradient boosting | Full NHANES population | Area under the ROC curve | 0.7289 |
| Gradient boosting | Full NHANES population | Average precision | 0.4845 |
| K-nearest neighbors | Curated holdout set | Accuracy | 1.0000 |
| K-nearest neighbors | Curated holdout set | F1-score | 1.0000 |
| K-nearest neighbors | Curated holdout set | Precision | 1.0000 |
| K-nearest neighbors | Curated holdout set | Recall | 1.0000 |
| K-nearest neighbors | Curated holdout set | Matthews correlation coefficient | 1.0000 |
| K-nearest neighbors | Curated holdout set | Area under the ROC curve | 1.0000 |
| K-nearest neighbors | Curated holdout set | Average precision | 1.0000 |
| K-nearest neighbors | Full NHANES population | Accuracy | 0.7202 |
| K-nearest neighbors | Full NHANES population | F1-score | 0.4996 |
| K-nearest neighbors | Full NHANES population | Precision | 0.4485 |
| K-nearest neighbors | Full NHANES population | Recall | 0.5638 |
| K-nearest neighbors | Full NHANES population | Matthews correlation coefficient | 0.3128 |
| K-nearest neighbors | Full NHANES population | Area under the ROC curve | 0.7065 |
| K-nearest neighbors | Full NHANES population | Average precision | 0.4186 |
| Gaussian naive Bayes | Curated holdout set | Accuracy | 0.9910 |
| Gaussian naive Bayes | Curated holdout set | F1-score | 0.9910 |
| Gaussian naive Bayes | Curated holdout set | Precision | 0.9940 |
| Gaussian naive Bayes | Curated holdout set | Recall | 0.9880 |
| Gaussian naive Bayes | Curated holdout set | Matthews correlation coefficient | 0.9820 |
| Gaussian naive Bayes | Curated holdout set | Area under the ROC curve | 0.9968 |
| Gaussian naive Bayes | Curated holdout set | Average precision | 0.9938 |
| Gaussian naive Bayes | Full NHANES population | Accuracy | 0.6820 |
| Gaussian naive Bayes | Full NHANES population | F1-score | 0.4879 |
| Gaussian naive Bayes | Full NHANES population | Precision | 0.4058 |
| Gaussian naive Bayes | Full NHANES population | Recall | 0.6115 |
| Gaussian naive Bayes | Full NHANES population | Matthews correlation coefficient | 0.2827 |
| Gaussian naive Bayes | Full NHANES population | Area under the ROC curve | 0.6885 |
| Gaussian naive Bayes | Full NHANES population | Average precision | 0.3767 |
| Support vector machine | Curated holdout set | Accuracy | 0.9990 |
| Support vector machine | Curated holdout set | F1-score | 0.9990 |
| Support vector machine | Curated holdout set | Precision | 0.9980 |
| Support vector machine | Curated holdout set | Recall | 1.0000 |
| Support vector machine | Curated holdout set | Matthews correlation coefficient | 0.9980 |
| Support vector machine | Curated holdout set | Area under the ROC curve | 1.0000 |
| Support vector machine | Curated holdout set | Average precision | 1.0000 |
| Support vector machine | Full NHANES population | Accuracy | 0.6703 |
| Support vector machine | Full NHANES population | F1-score | 0.5019 |
| Support vector machine | Full NHANES population | Precision | 0.4010 |
| Support vector machine | Full NHANES population | Recall | 0.6706 |
| Support vector machine | Full NHANES population | Matthews correlation coefficient | 0.2987 |
| Support vector machine | Full NHANES population | Area under the ROC curve | 0.7284 |
| Support vector machine | Full NHANES population | Average precision | 0.4819 |
| XGBoost | Curated holdout set | Accuracy | 0.9990 |
| XGBoost | Curated holdout set | F1-score | 0.9990 |
| XGBoost | Curated holdout set | Precision | 0.9980 |
| XGBoost | Curated holdout set | Recall | 1.0000 |
| XGBoost | Curated holdout set | Matthews correlation coefficient | 0.9980 |
| XGBoost | Curated holdout set | Area under the ROC curve | 1.0000 |
| XGBoost | Curated holdout set | Average precision | 1.0000 |
| XGBoost | Full NHANES population | Accuracy | 0.7019 |
| XGBoost | Full NHANES population | F1-score | 0.5036 |
| XGBoost | Full NHANES population | Precision | 0.4286 |
| XGBoost | Full NHANES population | Recall | 0.6103 |
| XGBoost | Full NHANES population | Matthews correlation coefficient | 0.3093 |
| XGBoost | Full NHANES population | Area under the ROC curve | 0.7251 |
| XGBoost | Full NHANES population | Average precision | 0.4687 |
| This table reports curated-holdout and full-population performance for the primary gradient-boosting model, logistic regression, random forest, gradient boosting, k-nearest neighbors, Gaussian naive Bayes, support vector machine, and XGBoost when available.Comparator-model classification metrics in this table were calculated at a common 0.50 probability threshold; the primary operating-point metrics in Tables 2 and 4 used the validation-selected threshold. | | | |

**Table S3.** Native, permutation, and SHAP-based feature-importance analyses.

| Feature | Native importance | Relative native importance | Importance method | Original feature | Standardized feature label | Permutation importance mean | Permutation importance SD | Mean absolute SHAP value |
| --- | --- | --- | --- | --- | --- | --- | --- | --- |
| Usual sleep duration | 280.0000 | 0.2228 | Native model importance |  |  |  |  |  |
| Total daily fat intake | 176.0000 | 0.1400 | Native model importance |  |  |  |  |  |
| Age | 128.0000 | 0.1018 | Native model importance |  |  |  |  |  |
| Daytime sleepiness frequency: Almost always: 16-30 times per month | 102.0000 | 0.0811 | Native model importance |  |  |  |  |  |
| Self-reported trouble sleeping: Yes | 97.0000 | 0.0772 | Native model importance |  |  |  |  |  |
| Daytime sleepiness frequency: Often: 5-15 times per month | 92.0000 | 0.0732 | Native model importance |  |  |  |  |  |
| Educational attainment: College graduate or above | 79.0000 | 0.0628 | Native model importance |  |  |  |  |  |
| Sex: Male | 67.0000 | 0.0533 | Native model importance |  |  |  |  |  |
| Total daily energy intake | 59.0000 | 0.0469 | Native model importance |  |  |  |  |  |
| Daytime sleepiness frequency: Never | 50.0000 | 0.0398 | Native model importance |  |  |  |  |  |
| Educational attainment: 9th-11th grade, including 12th grade without diploma | 33.0000 | 0.0263 | Native model importance |  |  |  |  |  |
| Self-reported trouble sleeping: No | 26.0000 | 0.0207 | Native model importance |  |  |  |  |  |
| Daytime sleepiness frequency: Rarely: 1 time per month | 20.0000 | 0.0159 | Native model importance |  |  |  |  |  |
| Sex: Female | 20.0000 | 0.0159 | Native model importance |  |  |  |  |  |
| Educational attainment: Less than 9th grade | 15.0000 | 0.0119 | Native model importance |  |  |  |  |  |
| Educational attainment: Some college or associate degree | 10.0000 | 0.0080 | Native model importance |  |  |  |  |  |
| Daytime sleepiness frequency: Sometimes: 2-4 times per month | 3.0000 | 0.0024 | Native model importance |  |  |  |  |  |
| Educational attainment: Missing or unavailable | 0.0000 | 0.0000 | Native model importance |  |  |  |  |  |
| Educational attainment: High school graduate/GED or equivalent | 0.0000 | 0.0000 | Native model importance |  |  |  |  |  |
|  |  |  | Permutation importance | slq050 | Self-reported trouble sleeping | 0.1865 | 0.0105 |  |
|  |  |  | Permutation importance | slq120 | Daytime sleepiness frequency | 0.0063 | 0.0007 |  |
|  |  |  | Permutation importance | sleep_hours | Usual sleep duration | 0.0012 | 0.0004 |  |
|  |  |  | Permutation importance | dmdeduc2 | Educational attainment | 0.0000 | 0.0000 |  |
|  |  |  | Permutation importance | riagendr | Sex | 0.0000 | 0.0000 |  |
|  |  |  | Permutation importance | ridageyr | Age | 0.0000 | 0.0000 |  |
|  |  |  | Permutation importance | dr1ikcal | Total daily energy intake | 0.0000 | 0.0000 |  |
|  |  |  | Permutation importance | dr1itfat | Total daily fat intake | 0.0000 | 0.0000 |  |
| Self-reported trouble sleeping: Yes |  |  | Global SHAP importance |  |  |  |  | 5.3964 |
| Self-reported trouble sleeping: No |  |  | Global SHAP importance |  |  |  |  | 1.2696 |
| Educational attainment: College graduate or above |  |  | Global SHAP importance |  |  |  |  | 0.8741 |
| Sex: Male |  |  | Global SHAP importance |  |  |  |  | 0.7883 |
| Usual sleep duration |  |  | Global SHAP importance |  |  |  |  | 0.6157 |
| Daytime sleepiness frequency: Often: 5-15 times per month |  |  | Global SHAP importance |  |  |  |  | 0.5217 |
| Daytime sleepiness frequency: Almost always: 16-30 times per month |  |  | Global SHAP importance |  |  |  |  | 0.4414 |
| Daytime sleepiness frequency: Never |  |  | Global SHAP importance |  |  |  |  | 0.3040 |
| Educational attainment: 9th-11th grade, including 12th grade without diploma |  |  | Global SHAP importance |  |  |  |  | 0.1449 |
| Total daily fat intake |  |  | Global SHAP importance |  |  |  |  | 0.1194 |
| Age |  |  | Global SHAP importance |  |  |  |  | 0.1182 |
| Daytime sleepiness frequency: Rarely: 1 time per month |  |  | Global SHAP importance |  |  |  |  | 0.1063 |
| Sex: Female |  |  | Global SHAP importance |  |  |  |  | 0.0887 |
| Educational attainment: Some college or associate degree |  |  | Global SHAP importance |  |  |  |  | 0.0474 |
| Total daily energy intake |  |  | Global SHAP importance |  |  |  |  | 0.0264 |
| Daytime sleepiness frequency: Sometimes: 2-4 times per month |  |  | Global SHAP importance |  |  |  |  | 0.0173 |
| Educational attainment: Less than 9th grade |  |  | Global SHAP importance |  |  |  |  | 0.0158 |
| Educational attainment: Missing or unavailable |  |  | Global SHAP importance |  |  |  |  | 0.0000 |
| Educational attainment: High school graduate/GED or equivalent |  |  | Global SHAP importance |  |  |  |  | 0.0000 |
| This table reports model-derived native importance, permutation importance, and global SHAP importance using standardized predictor and category labels. | | | | | | | | |

**Table S4.** Calibration deciles in the curated holdout set and full NHANES population.

| bin | observed | expected | n | Expected non event | Observed non event | Observed event rate | Mean predicted probability | Sample |
| --- | --- | --- | --- | --- | --- | --- | --- | --- |
| (3.37928e-05, 4.25854e-05] | 0 | 0.0040 | 103 | 102.9960 | 103 | 0.0000 | 0.0000 | Curated holdout set |
| (4.25854e-05, 6.67341e-05] | 0 | 0.0055 | 97 | 96.9945 | 97 | 0.0000 | 0.0001 | Curated holdout set |
| (6.67341e-05, 0.00011608] | 0 | 0.0086 | 100 | 99.9914 | 100 | 0.0000 | 0.0001 | Curated holdout set |
| (0.00011608, 0.000179593] | 0 | 0.0152 | 100 | 99.9848 | 100 | 0.0000 | 0.0002 | Curated holdout set |
| (0.000179593, 0.522995] | 0 | 0.1600 | 100 | 99.8400 | 100 | 0.0000 | 0.0016 | Curated holdout set |
| (0.522995, 0.999861] | 100 | 99.8743 | 100 | 0.1257 | 0 | 1.0000 | 0.9987 | Curated holdout set |
| (0.999861, 0.999902] | 100 | 99.9893 | 100 | 0.0107 | 0 | 1.0000 | 0.9999 | Curated holdout set |
| (0.999902, 0.999934] | 100 | 99.9921 | 100 | 0.0079 | 0 | 1.0000 | 0.9999 | Curated holdout set |
| (0.999934, 0.999946] | 101 | 100.9941 | 101 | 0.0059 | 0 | 1.0000 | 0.9999 | Curated holdout set |
| (0.999946, 0.999949] | 99 | 98.9948 | 99 | 0.0052 | 0 | 1.0000 | 0.9999 | Curated holdout set |
| (2.4792999999999998e-05, 9.8274e-05] | 277 | 0.2509 | 3797 | 3796.7491 | 3520 | 0.0730 | 0.0001 | Full NHANES population |
| (9.8274e-05, 0.00018633] | 429 | 0.5536 | 3795 | 3794.4464 | 3366 | 0.1130 | 0.0001 | Full NHANES population |
| (0.00018633, 0.00045796] | 515 | 1.0713 | 3801 | 3799.9287 | 3286 | 0.1355 | 0.0003 | Full NHANES population |
| (0.00045796, 0.0017487] | 527 | 3.6443 | 3791 | 3787.3557 | 3264 | 0.1390 | 0.0010 | Full NHANES population |
| (0.0017487, 0.0063496] | 684 | 13.2021 | 3803 | 3789.7979 | 3119 | 0.1799 | 0.0035 | Full NHANES population |
| (0.0063496, 0.051953] | 782 | 70.5632 | 3788 | 3717.4368 | 3006 | 0.2064 | 0.0186 | Full NHANES population |
| (0.051953, 0.94696] | 1000 | 1987.4316 | 3796 | 1808.5684 | 2796 | 0.2634 | 0.5236 | Full NHANES population |
| (0.94696, 0.9995] | 1368 | 3803.5594 | 3836 | 32.4406 | 2468 | 0.3566 | 0.9915 | Full NHANES population |
| (0.9995, 0.99989] | 1592 | 3762.0438 | 3763 | 0.9562 | 2171 | 0.4231 | 0.9997 | Full NHANES population |
| (0.99989, 0.99995] | 2228 | 3788.7093 | 3789 | 0.2907 | 1561 | 0.5880 | 0.9999 | Full NHANES population |
| This table reports observed event rates and mean predicted probabilities across predicted-risk deciles for the curated holdout set and full NHANES population. | | | | | | | | |

**Table S5.** Full-population subgroup performance by sex, educational attainment, and age group.

| Subgroup variable | Level | N | Positive N | Accuracy | F1-score | Precision | Recall | Matthews correlation coefficient | Area under the ROC curve |
| --- | --- | --- | --- | --- | --- | --- | --- | --- | --- |
| Sex | Male | 18697 | 3764 | 0.7184 | 0.4515 | 0.3714 | 0.5757 | 0.2857 | 0.7175 |
| Sex | Female | 19262 | 5638 | 0.6380 | 0.5361 | 0.4290 | 0.7144 | 0.2921 | 0.7187 |
| Educational attainment | Missing or unavailable | 2226 | 557 | 0.6739 | 0.4525 | 0.3901 | 0.5386 | 0.2346 | 0.6623 |
| Educational attainment | Less than 9th grade | 3403 | 1009 | 0.6879 | 0.5346 | 0.4792 | 0.6046 | 0.3092 | 0.7139 |
| Educational attainment | 9th-11th grade, including 12th grade without diploma | 4936 | 1577 | 0.6345 | 0.5535 | 0.4539 | 0.7089 | 0.2877 | 0.7135 |
| Educational attainment | High school graduate/GED or equivalent | 8305 | 2208 | 0.6718 | 0.5192 | 0.4252 | 0.6667 | 0.3049 | 0.7197 |
| Educational attainment | Some college or associate degree | 10791 | 2750 | 0.6723 | 0.5161 | 0.4138 | 0.6858 | 0.3119 | 0.7226 |
| Educational attainment | College graduate or above | 8298 | 1301 | 0.7128 | 0.4044 | 0.2996 | 0.6218 | 0.2728 | 0.7233 |
| Age group | 40-59 years | 11704 | 3173 | 0.6734 | 0.5390 | 0.4366 | 0.7041 | 0.3281 | 0.7462 |
| Age group | <40 years | 14209 | 3466 | 0.6898 | 0.4865 | 0.4081 | 0.6024 | 0.2867 | 0.7081 |
| Age group | ≥60 years | 12046 | 2763 | 0.6673 | 0.4831 | 0.3753 | 0.6779 | 0.2919 | 0.7241 |
| This table reports accuracy, F1-score, precision, recall, Matthews correlation coefficient, and area under the ROC curve across prespecified demographic subgroups. | | | | | | | | | |

**Table S6.** Observed category frequencies and labels for categorical variables.

| Variable | Variable label | Observed code | Category label | N | Proportion |
| --- | --- | --- | --- | --- | --- |
| riagendr | Sex | 1 | Male | 18697 | 0.4926 |
| riagendr | Sex | 2 | Female | 19262 | 0.5074 |
| slq050 | Self-reported trouble sleeping | 1 | Yes | 9600 | 0.2529 |
| slq050 | Self-reported trouble sleeping | 2 | No | 28349 | 0.7468 |
| slq050 | Self-reported trouble sleeping | Missing | Missing | 10 | 0.0003 |
| slq120 | Daytime sleepiness frequency | 0 | Never | 5662 | 0.1492 |
| slq120 | Daytime sleepiness frequency | 1 | Rarely: 1 time per month | 5098 | 0.1343 |
| slq120 | Daytime sleepiness frequency | 2 | Sometimes: 2-4 times per month | 6821 | 0.1797 |
| slq120 | Daytime sleepiness frequency | 3 | Often: 5-15 times per month | 3407 | 0.0898 |
| slq120 | Daytime sleepiness frequency | 4 | Almost always: 16-30 times per month | 1643 | 0.0433 |
| slq120 | Daytime sleepiness frequency | Missing | Missing | 15328 | 0.4038 |
| dmdeduc2 | Educational attainment | 0 | Missing or unavailable | 2226 | 0.0586 |
| dmdeduc2 | Educational attainment | 1 | Less than 9th grade | 3403 | 0.0896 |
| dmdeduc2 | Educational attainment | 2 | 9th-11th grade, including 12th grade without diploma | 4936 | 0.1300 |
| dmdeduc2 | Educational attainment | 3 | High school graduate/GED or equivalent | 8305 | 0.2188 |
| dmdeduc2 | Educational attainment | 4 | Some college or associate degree | 10791 | 0.2843 |
| dmdeduc2 | Educational attainment | 5 | College graduate or above | 8298 | 0.2186 |
| sddsrvyr | NHANES survey cycle | 4 | NHANES 2005-2006 | 4736 | 0.1248 |
| sddsrvyr | NHANES survey cycle | 5 | NHANES 2007-2008 | 5334 | 0.1405 |
| sddsrvyr | NHANES survey cycle | 6 | NHANES 2009-2010 | 5505 | 0.1450 |
| sddsrvyr | NHANES survey cycle | 7 | NHANES 2011-2012 | 4708 | 0.1240 |
| sddsrvyr | NHANES survey cycle | 8 | NHANES 2013-2014 | 5096 | 0.1343 |
| sddsrvyr | NHANES survey cycle | 9 | NHANES 2015-2016 | 4941 | 0.1302 |
| sddsrvyr | NHANES survey cycle | 66 | NHANES 2017-March 2020 pre-pandemic public release | 7639 | 0.2012 |
| This table reports observed category codes, standardized category labels, and frequencies for categorical variables. Nonstandard categories are flagged for verification before manuscript reporting. | | | | | |

**Table S7.** PHQ-9 distribution, outcome prevalence, and missingness after variable-specific cleaning.

| Section | Item | Value | Notes |
| --- | --- | --- | --- |
| PHQ-9 distribution | count | 37959.0000 | PHQ-9 total score after variable-specific cleaning |
| PHQ-9 distribution | mean | 3.2099 | PHQ-9 total score after variable-specific cleaning |
| PHQ-9 distribution | std | 4.2522 | PHQ-9 total score after variable-specific cleaning |
| PHQ-9 distribution | min | 0.0000 | PHQ-9 total score after variable-specific cleaning |
| PHQ-9 distribution | 25% | 0.0000 | PHQ-9 total score after variable-specific cleaning |
| PHQ-9 distribution | 50% | 2.0000 | PHQ-9 total score after variable-specific cleaning |
| PHQ-9 distribution | 75% | 4.0000 | PHQ-9 total score after variable-specific cleaning |
| PHQ-9 distribution | max | 27.0000 | PHQ-9 total score after variable-specific cleaning |
| Outcome threshold | PHQ-9 >=5 | 9402.0000 | Mild-or-greater depressive symptoms; prevalence=0.2477 |
| Outcome threshold | PHQ-9 >=10 | 3325.0000 | Moderate-or-greater depressive symptoms; prevalence=0.0876 |
| Primary predictor missingness | Self-reported trouble sleeping | 0.0003 | Missing proportion after variable-specific cleaning |
| Primary predictor missingness | Daytime sleepiness frequency | 0.4038 | Missing proportion after variable-specific cleaning |
| Primary predictor missingness | Sex | 0.0000 | Missing proportion after variable-specific cleaning |
| Primary predictor missingness | Educational attainment | 0.0000 | Missing proportion after variable-specific cleaning |
| Primary predictor missingness | Usual sleep duration | 0.0000 | Missing proportion after variable-specific cleaning |
| Primary predictor missingness | Total daily energy intake | 0.0000 | Missing proportion after variable-specific cleaning |
| Primary predictor missingness | Total daily fat intake | 0.0000 | Missing proportion after variable-specific cleaning |
| Primary predictor missingness | Age | 0.0000 | Missing proportion after variable-specific cleaning |
| Primary predictor missingness | PHQ-9 total score | 0.0000 | Missing proportion after variable-specific cleaning |
| This table reports the PHQ-9 total-score distribution, depressive-symptom threshold prevalence, and missingness for primary model variables after variable-specific cleaning. | | | |

**Figure S1.** Native LightGBM feature importance. This figure displays model-derived native feature importance using standardized feature and category labels. Native importance should be interpreted alongside permutation and SHAP analyses because rankings may differ across methods.

**Figure S2.** SHAP dependence plot for self-reported trouble sleeping. This plot shows SHAP contributions for the one-hot encoded indicator of self-reported trouble sleeping. Because the predictor is binary, points cluster at 0 and 1; positive SHAP values indicate greater contribution toward classification as mild-or-greater depressive symptoms.

**Figure S3.** Local SHAP explanation for the highest predicted-risk false-positive case. The plot shows the largest local SHAP contributions for the full-population participant with the highest predicted probability among participants who did not meet the PHQ-9 ≥5 outcome threshold. Positive SHAP values increased the model output toward classification as mild-or-greater depressive symptoms. The dominant positive contributors were self-reported trouble sleeping, almost-always daytime sleepiness, and usual sleep duration. Because categorical predictors were one-hot encoded, SHAP values represent contributions of encoded category indicators after translation back to readable NHANES response labels.

**Figure S4.** Confusion matrices for the curated holdout set and full NHANES population. The matrices show true-negative, false-positive, false-negative, and true-positive counts for the primary model in the curated holdout set and full NHANES population.

**Figure S5.** Cross-validated threshold selection and fold-level performance. This figure displays selected probability thresholds and fold-level classification performance in the curated corpus. These thresholds reflect internal validation behavior and should not be interpreted as clinical cut-points.
